# Local impedance and contact force guidance to predict successful cavotricuspid isthmus ablation with a zero-fluoroscopy approach

**DOI:** 10.3389/fcvm.2023.1322743

**Published:** 2024-01-04

**Authors:** Jorge Melero-Polo, Mercedes Cabrera-Ramos, Jose Manuel Alfonso-Almazán, Isabel Marín-García, Isabel Montilla-Padilla, José Ramón Ruiz-Arroyo, Guillermo López-Rodríguez, Javier Ramos-Maqueda

**Affiliations:** ^1^ Arrhythmias Unit, Department of Cardiology, Aragón Health Research Institute, University Hospital Clínico Lozano Blesa, Zaragoza, Spain; ^2^ Master of Science, Boston Scientific, Madrid, Spain

**Keywords:** cavotricuspid isthmus, catheter ablation, zero-fluoroscopy, local impedance, contact force

## Abstract

**Introduction:**

A new technology capable of monitoring local impedance (LI) and contact force (CF) has recently been developed. At the same time, there is growing concern regarding catheter ablation performed under fluoroscopy guidance, due to its harmful effects for both patients and practitioners. The aim of this study was to assess the safety and effectiveness of zero-fluoroscopy cavotricuspid isthmus (CTI) ablation monitoring LI drop and CF as well as to elucidate if these parameters can predict successful radiofrequency (RF) applications in CTI ablation.

**Methods:**

We conducted a prospective observational study recruiting 50 consecutive patients who underwent CTI ablation. A zero-fluoroscopy approach guided by the combination of LI drop and CF was performed. In each RF application, CF and LI drop were monitored. A 6-month follow-up visit was scheduled to assess recurrences.

**Results:**

A total of 767 first-pass RF applications were evaluated in 50 patients. First-pass effective RF applications were associated with greater LI drops: absolute LI drops (30.05 ± 6.23 Ω vs. 25.01 ± 5.95 Ω), *p* = 0.004) and relative LI drops (−23.3 ± 4.9% vs. −18.3 ± 5.6%, *p* = 0.0005). RF applications with a CF between 5 and 15 grams achieved a higher LI drop compared to those with a CF below 5 grams (29.4 ± 8.76 Ω vs. 24.8 ± 8.18 Ω, *p* < 0.0003). However, there were no significant differences in LI drop between RF applications with a CF between 5 and 15 grams and those with a CF beyond 15 grams (29.4 ± 8.76 Ω vs. 31.2 ± 9.81 Ω, *p* = 0.19). CF by itself, without considering LI drop, did not predict effective RF applications (12.3 ± 7.54 g vs. 11.18 ± 5.18 g, *p* = 0.545). Successful CTI ablation guided by a zero-fluoroscopy approach was achieved in all patients. Only one patient experienced a recurrence during the 6-month follow-up.

**Conclusions:**

LI drop (absolute and relative values) appears to be a good predictor of successful RF applications to achieve CTI conduction block. The optimal CF to achieve a good LI drop is between 5 and 15 g. A zero-fluoroscopy approach guided by LI and CF was feasible, effective, and safe.

## 1 Introduction

Radiofrequency catheter ablation (RFCA) along the CTI is considered the cornerstone of CTI-dependent atrial flutter treatment (1). The target of this technique is to achieve CTI-bidirectional block (BDB), which is associated with high long-term success rates. To guide RFCA procedures, some markers of lesion quality are frequently used to predict appropriate RF applications.

The most employed lesion markers are CF and a couple of indices such as ablation index (AI) and lesion index (LSI), both mainly based on a combination of RF time, power, and CF (2).

In the past few years, a new ablation catheter (Intellanav MiFi OI™, Boston Scientific, Marlborough, U.S.A) capable of measuring real-time LI and LI drop while applying RF has been developed. LI drop results from tissue heating and constitutes a good predictor of lesion formation, catheter-tissue coupling, and effective ablation in different arrhythmic substrates, such as atrial fibrillation (3) and CTI-dependent atrial flutters (4, 5). More recently, a new catheter with new technology and a different design (ie, without micro-electrodes) has been introduced (Intellanav StablePoint™, Boston Scientific, Marlborough, U.S.A), measuring not just LI drop but also assessing CF. Since this catheter measures LI using a novel technology, the cut-off values of LI drop have not yet been established for any RFCA procedure.

In recent years, there has been a growing concern about x-ray exposure during fluoroscopy-guided RFCA procedures, due to its harmful effects on both patients and operators (6). Given that there is a definite risk of cancer induction (7) and no minimum safe threshold dose, the radiation dose should be reduced to the lowest possible following the ALARA principle (As Low As Reasonably Achievable). As a matter of fact, the awareness of the ionizing radiation risks has led many electrophysiologists to erase the use of X-rays, by implementing zero-fluoroscopy procedures guided by electroanatomical navigation systems (8, 9) and more recently, intracardiac echocardiography (ICE) (10).

Several studies and large meta-analyses have proven the feasibility, efficacy and safety of a zero/minimal fluoroscopy approach during supraventricular arrhythmias RFCA, reducing radiation exposure and ablation time (8–12). This non-fluoroscopic workflow also benefits health care professionals, minimizing their accumulated radiation dose and reducing the risk of developing severe spinal injuries related to wearing lead aprons (13, 14).

In this study, our aim was to evaluate whether using this new technology capable of monitoring simultaneously both LI drop and CF could be useful in predicting appropriate RF lesions. Furthermore, we assessed the feasibility, safety, and efficacy of a zero-fluoroscopy CTI ablation guided by LI drop and CF.

## 2 Methods

### 2.1 Study population

We conducted a prospective observational single-center study including 767 RF applications in 50 consecutive patients who underwent CTI RFCA from January 2022 to January 2023. The study was conducted in full compliance with the Declaration of Helsinki and all patients included in the study signed an informed consent. Patients underwent clinical follow-up and ECG at 6-months post ablation.

### 2.2 Zero-fluoroscopy workflow

Because of the x-ray exposure risk, all the CTI ablations were performed with a fully zero-fluoroscopy approach, with the operator wearing no safety lead aprons. We evaluated the number of cases in which fluoroscopy was finally required due to technical difficulties or complications during the procedure (i.e., due to issues with the catheter access or during the ablation procedure, complications or lack of success). The procedures were performed by 2 operators, one of them with more than 500 zero-fluoroscopy ablations and the other one with less than 50 cases at the beginning of the study (27 and 23 cases respectively).

RFCA procedures were performed under conscious sedation. Our workflow included an echo-guided double femoral puncture. After that, the magnetically tracked Intellanav StablePoint™ irrigated catheter was gently introduced through the femoral access and guided to the right atrium (RA) by Rhythmia HDx mapping system (Boston Scientific, Marlborough, U.S.A). A RA electroanatomical map was performed and certain structures were tagged on it guided by EGM signals (i.e., tricuspid annulus, hisian region, coronary sinus ostium, and CTI superior and inferior borders). Subsequently, we introduced a fixed curve decapolar diagnostic catheter into the coronary sinus through the second femoral access.

### 2.3 Cavotricuspid isthmus ablation guided by contact force and local impedance

The CTI RFCA was guided by CF and DIRECTSENSE™ technology, a feature that confirms electrical contact and catheter tip-to-tissue stability and provides real-time data of LI. A point-by-point RFCA was conducted with open irrigation, from the tricuspid annulus (TA) to the inferior vena cava (IVC), with a maximal interlesion distance of 5 mm.

All our patients had previously developed CTI-dependent atrial flutter. In the electrophysiology room, 13 patients (26%) presented CTI-dependent atrial flutter, 3 patients (6%) atrial fibrillation and 34 patients (68%) were in sinus rhythm.

If the patient arrived to the electrophysiology room in sinus rhythm, the ablation was performed while pacing at 700 ms cycle length from the proximal dipole of a decapolar catheter placed at the coronary sinus (CS) to facilitate the visualization of BDB. In patients who arrived with an ongoing CTI-dependent atrial flutter, the ablation was initially performed during this rhythm, and after recovering sinus rhythm during ablation, we completed the ablation pacing from the coronary sinus catheter as described before. The patients with ongoing atrial fibrillation underwent electric cardioversion before starting the procedure to restore sinus rhythm, and then the ablation was performed following the same approach.

To start RF applications a minimum CF of 3 g was required and a maximum CF of 25 g was allowed. The maximum interlesion distance allowed was 5 mm and therefore no visual gaps were permitted. The targeted LI drop range was between 30 and 45 Ω. If a LI drop of 30–45 Ω was achieved in the first 30 s the RF application was considered successful and therefore stopped. If a LI drop of 30 Ω was not achieved RF application was prolonged up to 45 s. The RF application also was stopped if LI drop exceeded 45 Ω or if LI dropped abruptly exceeding 40 Ω in the first 5 s of the RF application. Thus, our approach was mainly based on LI drop with CF support, unlike classic approaches which usually are exclusively based on CF or some indices emanated from it (15, 16) or even without CF data, with 8 mm catheters in temperature regulated ablation (17).

To understand the role of the CF in the LI drop, we categorized the mean CF of each RF application into three clusters: 0–5 g, 5–15 g and 15–25 g. RF energy was applied in power control mode (fixed at 45 W) with a temperature limit of 43°C at an irrigation flow of 30 ml/min.

**Figure 1:**
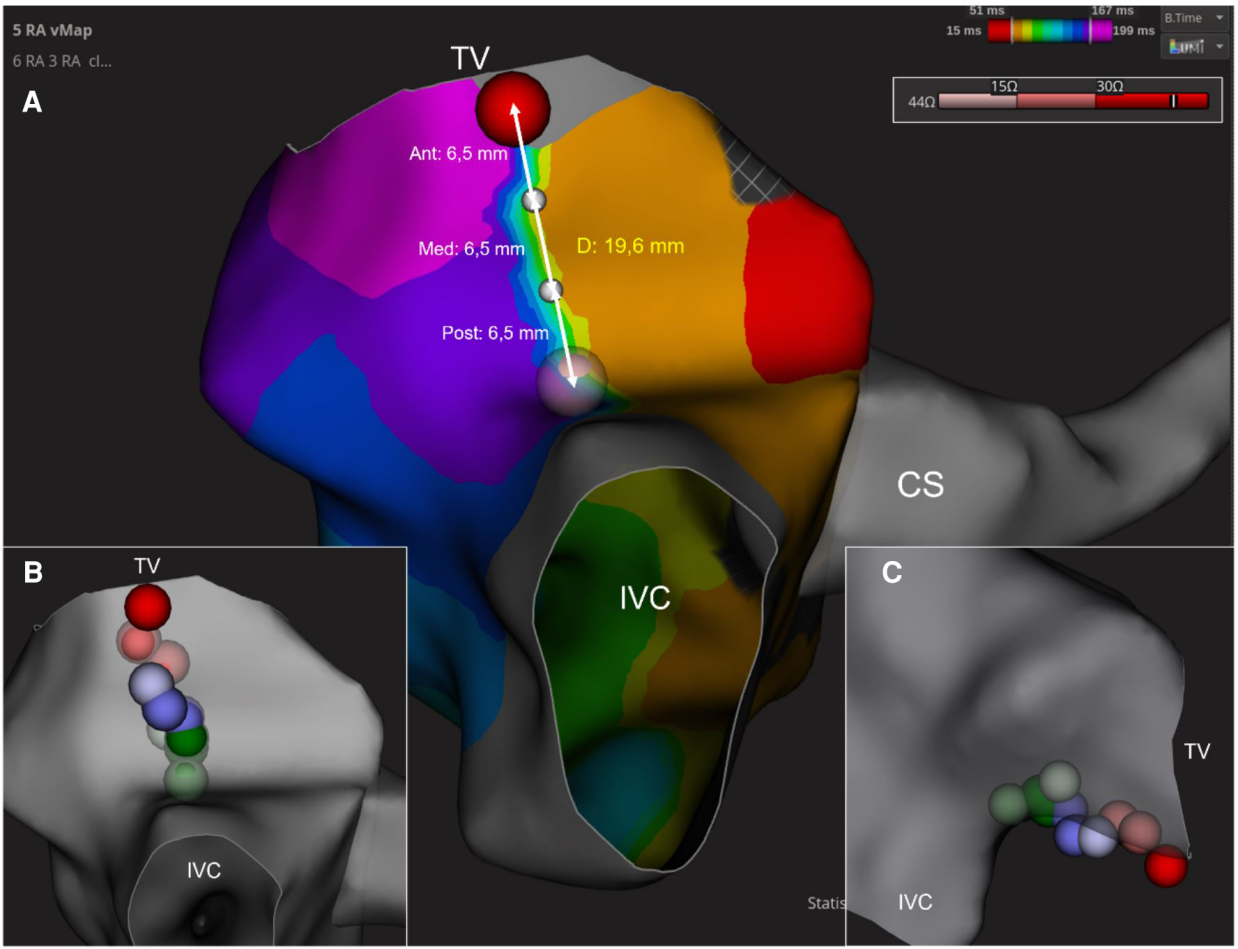
Figure shows how the cavotricuspid isthmus is divided into 3 equally-sized regions from the tricuspid valve to the inferior vena cava: anterior (ant), medium (med) and posterior (post) segments (**A**). RF applications were assigned to the corresponding region: red (anterior), blue (medium) and green (posterior) (**B,C**). CS, coronary sinus; CTI, cavotricuspid isthmus; IVC, inferior vena cava; TV, tricuspid valve.

A first CTI ablation line was performed from the TA to the IVC. When this line was completed, the presence of BDB was assessed by counterclockwise and clockwise activation maps pacing from the proximal CS or the ablation catheter (positioned laterally to the CTI line) respectively. If BDB was present after this first ablation line and persisted after a waiting time of 30 min (18), first-pass conduction block was established (5), and all RF applications were considered successful. If BDB was not present after the first ablation line or we detected and early recovery in conduction across the CTI during the waiting time, an activation map along the CTI line was performed from the TA to the IVC to localize the gap. To establish the gap location, the CTI was divided into three equally sized regions from the TA to the IVC: anterior, mid and posterior segments. The distance from the most anterior RF application to the most posterior one was calculated and divided into 3 equal parts, so the gaps could be linked to one of these 3 regions (Figure 1). When a gap was detected, the corresponding segment was valued as a “gap segment” and the RF applications included in that segment were considered as ineffective. On the other hand, a RF application was considered successful if it belonged to a gap-free segment. This classification approach was adopted because accurately locating the gap in the line can often be imprecise without using Ultra-High density mapping. Therefore, it can be difficult to distinguish which one of the adjacent RF applications is the one that had been ineffective (19).

When a gap was localized, its position was tagged and extra RF applications were performed in that location until BDB was achieved. These additional lesions were excluded from the RF applications analysis, which only evaluated first-pass applications (the first line of RF applications from the TA to the IVC) to avoid a potential bias in LI drop by applying RF to tissue that had already been treated previously (20).

### 2.4 Clinical outcomes

We aimed to achieve the following objectives: (i) to assess CTI RFCA guided by LI and CF acute success; (ii) to evaluate the role of LI drop and CF predicting effective lesions; (iii) To analyze the safety and effectiveness of a zero-fluoroscopy approach using the electroanatomic navigation system Rhythmia and the DIRECTSENSE™ technology and (iv) to consider CTI dependent atrial flutter recurrences at 6 months follow up with this strategy. Recurrence was defined as any documented arrythmia suggestive of CTI-dependent atrial flutter during the follow-up. In case of palpitations with no ECG evidence, we offered our patients a home ECG monitor to rule out recurrences.

### 2.5 Statistical analysis

Continuous variables were expressed as the mean ± standard deviation (SD). Variable normality was tested using Shapiro-Wilk test. A *χ*^2^ test was used to compare categorical variables. For comparison of continuous variables between two groups, Student's *t*-test or Mann-Whitney *U*-test were used depending on whether the data followed a normal distribution or not.

Comparisons of more than two groups were conducted using one-way ANOVA. For comparisons involving multiple factors, the two-way ANOVA was employed. The correlation between continuous variables was evaluated using Pearson's correlation coefficient. The statistical significance was considered at a *p* value <0.05. The statistical analyses were performed using RStudio (2022.07.2).

## 3 Results

### 3.1 Radiofrequency catheter ablation

Table 1 presents the baseline characteristics of the 50 patients included in the analyses. The mean age was 65.59 ± 9.48 years and 76% of the patients were male. A total of 767 lesions were analyzed in 50 patients who underwent a CTI RFCA (15.3 ± 4.78 lesions per patient). Acute success was achieved in 50 (100%) patients. First-pass CTI BDB was achieved in 35 patients (70.0%), while 15 patients (30%) exhibited a conduction gap after first-pass ablation. In 2 (12.5%) of these patients the gap was located in the anterior portion of the CTI, in 7 (43.7%), in the mid-segment and in another 7 (43.7%) in the posterior part (one patient had two gaps, in the mid and anterior segments).

**Table 1:** Baseline characteristics and procedure parameters. Results are shown in mean ± standard deviation or *n* (%).

Parameters	Value
*n*	50 (100)
Age (year)	65.90 ± 9.48
Male sex	38 (76)
Hypertension	23 (46)
Diabetes mellitus	12 (24)
History of heart failure	6 (12)
CHA2DS2-VASc score	2.10 ± 1.38
LVEF (%)	55.03 ± 13.52
Structural heart disease	10 (20)
History of atrial fibrillation	18 (36)
Total procedure time (min)	92 ± 17
Total ablation time (min)	26 ± 9
Successful CTI ablation	50 (100)

LVEF, left ventricular ejection fraction; CTI, cavotricuspid Isthmus.

### 3.2 Local impedance and contact force

**Figure 2:**
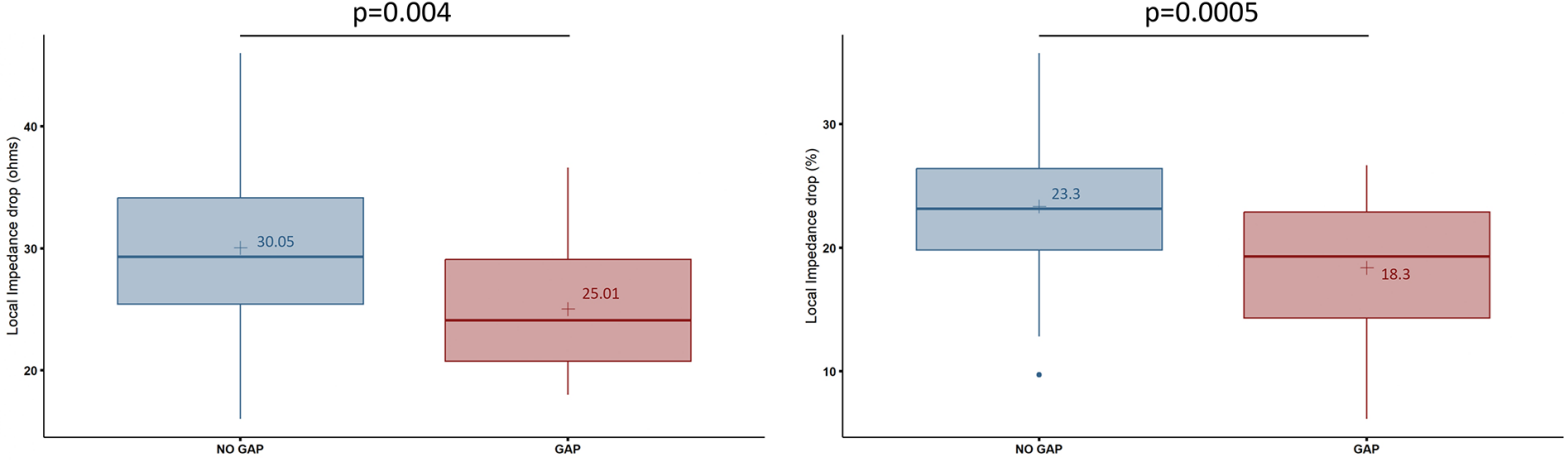
Local impedance drop in absolute values (Ω, left) and relative values (percentage, right) at the segments with and without conduction gaps during CTI ablation. In the box plots, the horizontal lines with boxes and bars represent the median with interquartile range and maximum and minimum values, and the numerical value (+) corresponds to the mean value.

**Figure 3:**
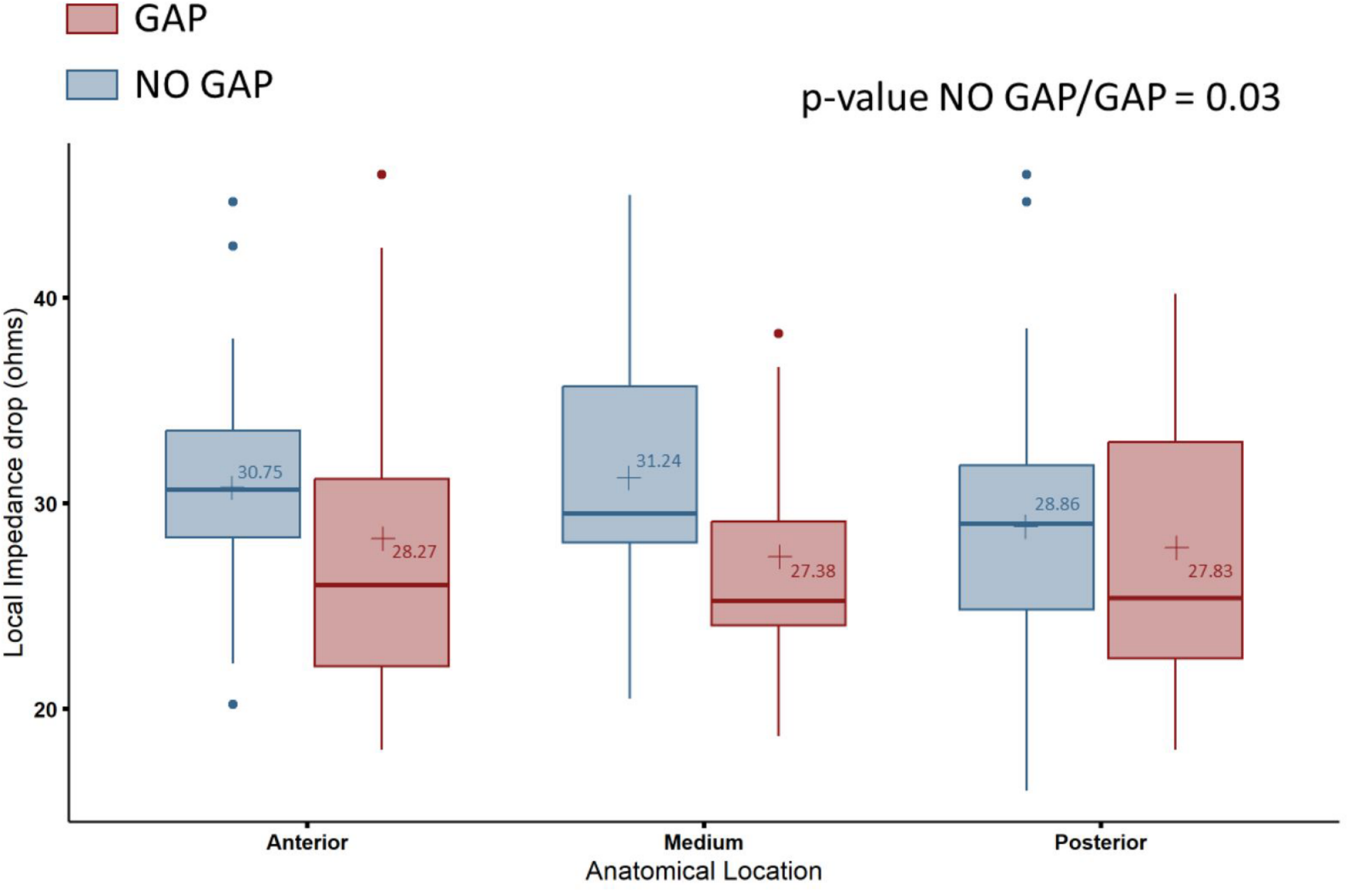
Comparison of local impedance drop in absolute values (Ω) at the segments with and without conduction gaps in the different CTI segments: anterior, medium and posterior. CTI, cavotricuspid isthmus. In the box plots, the horizontal lines with boxes and bars represent the median with interquartile range and maximum and minimum values, and the numerical value (+) corresponds with the mean value.

The segments without conduction gaps were associated with greater absolute LI drops (30.05 ± 6.23 Ω vs. 25.01 ± 5.95 Ω, *p* = 0.004) and relative LI drops (−23.3 ± 4.9% vs. −18.3 ± 5.6%, *p* = 0.0005) compared with those segments with conduction gaps (Figure 2). This association was independent of the location within any of the 3 segments (*p* = 0.208) (Figure 3). The optimal cutoff value of LI drop to predict effective ablation was 24.27 Ω, with a sensitivity and specificity of 64.3% and 82.1% respectively. In the ROC analysis, an area under the curve (AUC) of 0.73 (0.569–0.885) was observed.

Initial LI values before ablation did not differ between effective and ineffective ablation segments *(*164 Ω ± 29.8 vs. 161 Ω ± 13.2, *p* = 0.69). However, a correlation between initial local impedance and LI drop was found (*r* = 0.54, *p* = 0.0001).

**Figure 4:**
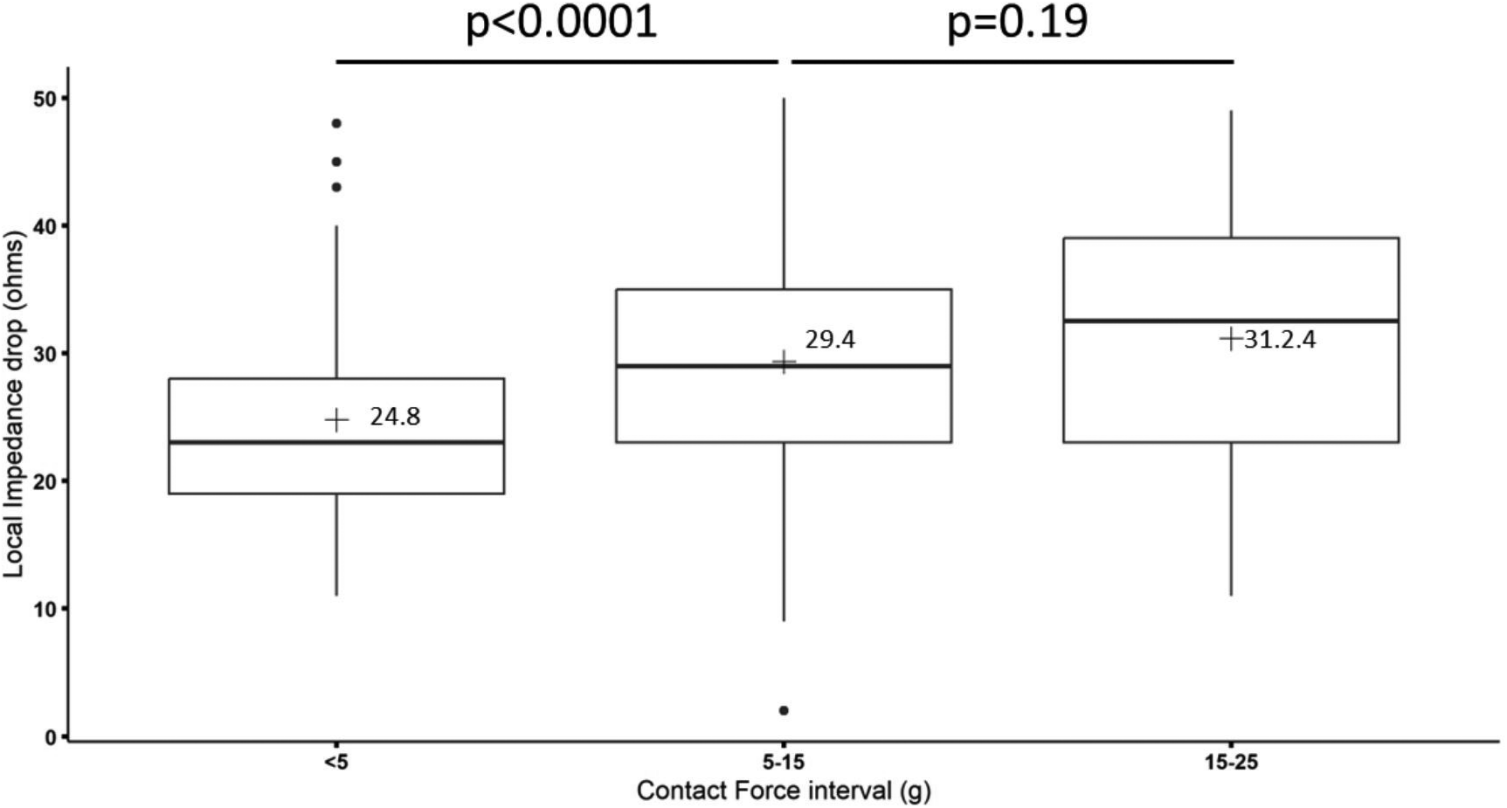
Relationship between local impedance drop in absolute values (Ω) and contact force (g) during CTI radiofrequency ablation. In the box plots, the horizontal lines with boxes and bars represent the median with interquartile range and maximum and minimum values, and the numerical value (+) corresponds with the mean value.

In terms of the relationship between LI and CF, RF applications were divided into three groups depending on the CF present in each application. When a CF between 5 and 15 g was present, a higher LI drop was achieved compared to those with a CF below 5 g (29.4 ± 8.76 Ω vs. 24.8 ± 8.18 Ω, *p* < 0.0003). However, there were no significant differences in LI drop between RF applications with a CF between 5 and 15 g and those with a CF beyond 15 g (29.4 ± 8.76 Ω vs. 31.2 ± 9.81 Ω, *p* = 0.19) (Figure 4). The CF itself without considering LI drop did not predict effective RF applications (12.3 ± 7.54 g vs. 11.18 ± 5.18 g, *p* = 0.545).

### 3.3 Safety and effectiveness of a zero-fluoroscopy cavotricuspid isthmus ablation

A successful zero fluoroscopy procedure was achieved in every patient with no complications registered. The operators did not have to convert the procedure to a fluoroscopy-guided ablation in any patient. Intracardiac echocardiography (ICE) was available at the discretion of the operators, but its use was not necessary in any case. After a median follow-up period of 6 months, only one patient (2%) experienced a recurrence.

## 4 Discussion

### 4.1 Major findings

This study drew three key findings: (i) the LI drop was higher in those segments without conduction gaps; (ii); when the CF was between 5 and 15 g, a higher LI drop was achieved compared to when it was <5 g; however, there were no additional benefits when applying with a CF > 15 g; (iii) a zero-fluoroscopy approach using this new technology was feasible, effective and safe.

### 4.2 Contact force and local impedance monitoring during ablation

CTI RFCA is currently the gold standard therapy for CTI-dependent flutters. BDB has been established for many years as the best endpoint for achieving excellent long-term outcomes (21). However, the heterogeneity of CTI anatomy, characterized by frequent trabeculations and recesses, can make difficult to determine if an application has been effective. In this regard, CF is a widely used parameter to predict the effectiveness of the lesion during ablation procedures. It refers to the measurement of the force applied by the ablation catheter on the tissue during the procedure. By monitoring CF, optimal tissue contact can be ensured, and the appropriate energy can be delivered to create a successful lesion. It allows for applications with greater stability and, therefore, more efficiency (22).

While CF has been shown to predict effective lesions in pulmonary vein isolation in the treatment of atrial fibrillation (23, 24), the data on CTI ablation remain controversial. While some observational studies showed a reduction in the procedural and ablation times (22, 25), two randomized trials (15, 16) concluded that CF-guided ablation did not reduce re-conduction across the CTI after 3 months neither reduced fluoroscopy, ablation, or total procedure times. In the same line, Pang et al. recently demonstrated in a meta-analysis that CF does not improve the acute success rate and long-term outcomes (26).

Hence, other variables are necessary to predict successful lesion during CTI ablation. In recent years, LI has emerged as a new parameter capable of characterizing tissue composition, catheter-tissue coupling, and predicting lesion diameter and depth in real-time (20, 27). Furthermore, it has been proven that LI drop can predict effective lesions in atrial fibrillation ablation (28). LI measurement was first tested with a 4.5 mm tip ablation catheter with micro-electrodes (Intellanav MiFi OI™, Boston Scientific, Marlborough, U.S.A). LI drop measured with this catheter proved to be a good marker of lesion effectiveness in CTI ablation (4, 19, 29). Currently, a new catheter capable of measuring at the same time LI and CF is available (Intellanav StablePoint™, Boston Scientific, Marlborough, U.S.A). As the way these 2 catheters measure LI is not the same, the drop in LI is not transposable from the first to the latter.

Only one previous study has assessed the usefulness of combining LI and CF to predict success in CTI ablation. Sasaki et al. (5) found that a greater LI drop was associated with an effective lesion. Our results are consistent with this finding. Furthermore, our work provides additional information regarding this article: (i) RF was applied in power control mode, with higher power than the previous study (fixed in 45 W instead of 25–40 W). This could explain why in our work, the mean absolute LI drop in effective sites is noticeably higher than in the latter (30 vs. 22 Ω). Applying RF guided by LI was safe; indeed, none of our patients presented audible steam pops, cardiac tamponade or any other complication. It is worth mentioning that in the present study, all the included patients had previously developed common atrial flutter, in contrast to the patients included in the study of Sasaki et al. (5) (they reported only 22% of their cohort having suffered CTI-dependent Flutters prior to ablation), that is to say they performed prophylactic CTI ablation in the majority of their patients. Given the profile of patients included in the current research, a LI drop of 30 Ω is probably more appropriate than 22 Ω in patients undergoing a CTI ablation due to experiencing a common atrial flutter. The totality of the patients included in our investigation presented common atrial flutter, which leads to a greater structural remodeling and fibrosis, and therefore larger right atria, who are currently the candidates for CTI ablation according to guidelines. This point could also explain that the CTI ablation time in our study is longer and brings special relevance to our clinical outcomes (with just one recurrence of CTI-dependent flutter in the mid-term). As only 22% of patients of the previous study had developed CTI-dependent flutters previously to the ablation, its low recurrence rate after a 6-month follow-up is expected.

A recent study analyzing the usefulness of simultaneously measuring LI and CF during AF ablation proved that a higher CF was associated with an increased likelihood of ideal LI drop and that CF greater than 25 g did not have a major impact on LI drop (30). To the best of our knowledge, the current study is the first one to demonstrate that in CTI ablation the optimal CF to achieve an adequate LI drop is 5–15 g, achieving a greater LI drop than with a CF < 5 g and reaching no additional benefits in terms of LI drop with a CF > 15 g.

On the basis of the results of this research, LI is a good success predictor in RFCA CTI-ablation, and CF is a valuable parameter, which leads to achieving larger LI drops with CF > 5 g and consequently it is helpful to achieve CTI BDB. Therefore, we firmly believe that this new technology based on both, LI and CF can provide added value in CTI ablation. Thus, we propose targeting CF between 5 and 15 g and a LI drop of around 30 Ω to accomplish a successful and safe CTI ablation using the DIRECTSENSE™ technology. However, larger and randomized studies are necessary to find the optimal CF and LI values for a more efficient and improved CTI ablation guided by LI and CF.

Furthermore, considering the significant risk associated with exposure to ionizing radiation and its health implications (6), zero-fluoroscopy procedures should be encouraged. In CTI ablation, a non-fluoroscopic approach guided by three-dimensional navigation system has proven to be feasible, effective and safe (9). In the last years, guidance by ICE has demonstrated reducing not only fluoroscopy times but also ablation and procedure times (31) and allowing successful ablation in some challenging cases (32). More recently, zero-fluoroscopy guided exclusively by ICE has been described in two studies (10, 33) as an effective and safe approach, without impacting procedural times and helping to recognize early potential complications. Thus, ICE could constitute a valuable alternative for zero-fluoroscopy CTI ablation, but further and larger studies are needed to confirm these preliminary findings.

Finally, we believe that the present study has a significant added value. That is because although Rhythmia navigation system has proven to be useful in zero-fluoroscopy workflows in different types of catheter ablations (34, 35), this is the first research proving the safety and efficacy of a zero-fluoroscopy approach using Rhythmia in CTI ablation.

### 4.3 Limitations

The number of patients included in the study was modest, although sufficient to draw significant conclusions, especially considering the lack of experience in CTI-ablation combining LI and CF. The targeted LI was determined taking into account LI drop in the left atrium for AF ablation. In relation to the way the presence of gaps was established (i.e., considering segments), this work could have been more accurate if the gaps presence had been established based on high-density mapping catheters. However, being aware of this limitation, the study results are significant, and we believe that by addressing this limitation, the results would be even more significant.

## 5 Conclusions

LI drop (absolute and relative values) appears to be a good predictor of successful radiofrequency applications in CTI ablation. The optimal CF to reach a good LI drop is between 5 and 15 g. However, the CF by itself, without considering LI drop, did not predict effective RF applications. In addition, a zero-fluoroscopy approach guided by LI and CF was feasible, effective, and safe.

## Data Availability

The raw data supporting the conclusions of this article will be made available by the authors, without undue reservation.
